# Lack of adaptation to human tetherin in HIV-1 Group O and P

**DOI:** 10.1186/1742-4690-8-78

**Published:** 2011-09-28

**Authors:** Su Jung Yang, Lisa A Lopez, Colin M Exline, Kevin G Haworth, Paula M Cannon

**Affiliations:** 1 Department of Molecular Microbiology and Immunology, Keck School of Medicine of the University of Southern California, Los Angeles, California, USA

## Abstract

**Background:**

HIV-1 viruses are categorized into four distinct groups: M, N, O and P. Despite the same genomic organization, only the group M viruses are responsible for the world-wide pandemic of AIDS, suggesting better adaptation to human hosts. Previously, it has been reported that the group M Vpu protein is capable of both down-modulating CD4 and counteracting BST-2/tetherin restriction, while the group O Vpu cannot antagonize tetherin. This led us to investigate if group O, and the related group P viruses, possess functional anti-tetherin activities in Vpu or another viral protein, and to further map the residues required for group M Vpu to counteract human tetherin.

**Results:**

We found a lack of activity against human tetherin for both the Vpu and Nef proteins from group O and P viruses. Furthermore, we found no evidence of anti-human tetherin activity in a fully infectious group O proviral clone, ruling out the possibility of an alternative anti-tetherin factor in this virus. Interestingly, an activity against primate tetherins was retained in the Nef proteins from both a group O and a group P virus. By making chimeras between a functional group M and non-functional group O Vpu protein, we were able to map the first 18 amino acids of group M Vpu as playing an essential role in the ability of the protein to antagonize human tetherin. We further demonstrated the importance of residue alanine-18 for the group M Vpu activity. This residue lies on a diagonal face of conserved alanines in the TM domain of the protein, and is necessary for specific Vpu-tetherin interactions.

**Conclusions:**

The absence of human specific anti-tetherin activities in HIV-1 group O and P suggests a failure of these viruses to adapt to human hosts, which may have limited their spread.

## Background

Tetherin (BST-2/CD317/HM1.24) is an interferon-inducible plasma membrane protein that can inhibit the release of enveloped viruses by physical tethering nascent virions at the cell surface [1,2]. Within the primate lentiviruses, this restriction is counteracted by anti-tetherin activities present in either the Vpu, Nef or Env proteins [1-11]. Several of these interactions are species-specific, suggesting that selection to evolve and maintain anti-tetherin functions has been part of the adaptation of the viruses to their hosts. For example, HIV-1 clade B Vpu counteracts human, but not primate or rodent tetherins [7,12,13], while the SIVmac and SIVcpz Nef proteins antagonize macaque and chimpanzee tetherin, but not the human protein [3-5,7].

**Figure 1:**
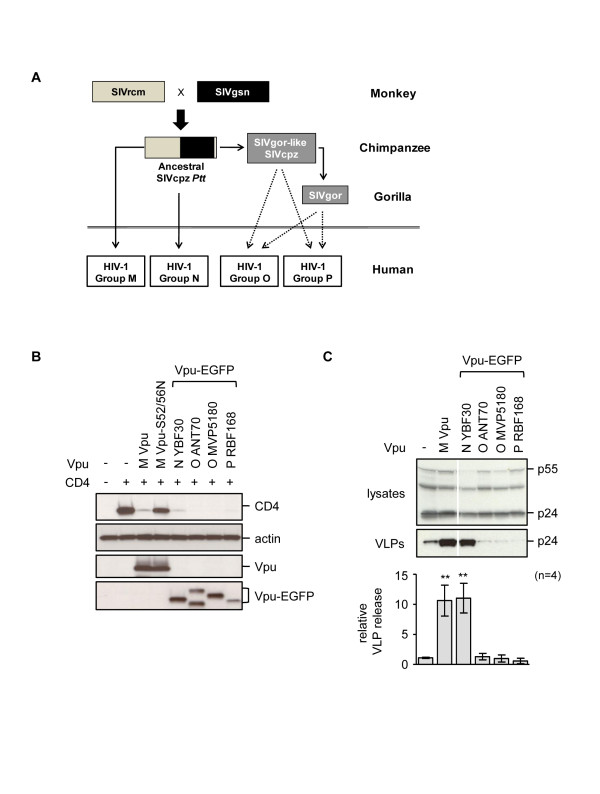
**Anti-tetherin activities of Vpu proteins from major HIV-1 groups**. **(A) **Origins of the four major groups of HIV-1. Solid arrows represent established transmissions, while broken arrows are more speculative events. **(B) **Ability of Vpu or Vpu-EGFP constructs to degrade CD4, examined by co-transfection of HeLa cells with a CD4 expression plasmid and the indicated Vpu plasmid. Western blots of cell lysates probed with the indicated antibodies are shown. The Vpu- constructs are described by both the HIV-1 group letter and the virus strain. As controls we included a group M Vpu from isolate NL4-3, and its S52/56N mutant that is unable to degrade CD4 [65]. **(C) **HIV-1 VLP release from tetherin-positive HeLa cells was measured by co-transfection of pHIV-1-pack (expresses HIV-1 Gag-Pol, Rev) in the absence (-) or presence of the indicated Vpu plasmids. VLP release was measured as the ratio of p24-reacting bands in the supernatants versus cell lysates following Western blot analysis, and made relative to the baseline level in the absence of Vpu, for n = 4 independent experiments. Statistical significance is indicated as *p *< 0.01 (**).

HIV-1 is classified into four distinct groups that maintain a similar genome organization but are highly divergent in their sequences: M (major), O (outlier), N (non-major, non-outlier), and P (putative) [14-17] (Figure 1A). Although all four groups of HIV-1 originated from the SIVcpz that infects *Pan troglodytes troglodytes *(*Ptt*) chimpanzees [18], they are interspersed among the present day SIVcpz *Ptt *lineages in distinct clusters, suggesting that each group arose by an independent ape to human transmission event [19,20]. HIV-1 groups M and N, and SIVcpz, are phylogenetically approximately equidistant from each other, while HIV-1 groups O and P are more closely related to the recently discovered SIVgor [17,18,21,22].

Overall, the independent cross-species transmission events that gave rise to the four known groups of HIV-1 have resulted in very different outcomes in terms of virus distribution [19]. HIV-1 group M is the most prevalent and diverse of the groups, accounting for greater than 90% of worldwide HIV-1 infections and driving the global pandemic of AIDS. In contrast, group N infections are very rare and have only been reported in a limited number of individuals in south central Cameroon [23-25]. HIV-1 group O is also rare, being mainly restricted to west central Africa [26,27] and accounting for 1% of infections in Cameroon [25,28,29]. Group P has been isolated from two individuals of Cameroonian descent [17,30].

It is unclear why the group M viruses have spread to become a global pandemic while the other viruses remain more limited in prevalence and geographical distribution. Although one study reported that HIV-1 O isolates may have reduced replicative fitness [31], a more recent study found comparable fitness, and similar or even higher cytopathicity when compared to group M isolates [32]. In addition, no major differences have been reported in the pathogenicity of group M and O infections [33,34], and the genetic diversity present in group O suggests that it is not a recent zoonotic transmission [16,35-37].

A previous study of anti-tetherin activities in HIV-1 groups M, N and O found that while the Vpu proteins from multiple group M and a single group N virus were able to antagonize human tetherin, no group O Vpu proteins had this activity [3]. In addition to targeting tetherin, Vpu also degrades CD4 complexed with HIV-1 Env in the endoplasmic reticulum [38-41] and all of the group O Vpu proteins were found to be able to reduce CD4 cell surface expression [3]. The Nef proteins from seven group O isolates were also evaluated, and none of these displayed activity against human tetherin [3]. These observations led us to question whether group O viruses have an anti-tetherin activity that is a function of a gene other than Vpu or Nef, or whether they are simply unable to counteract human tetherin, a feature that may have contributed to their limited penetration into human populations.

## Results

### HIV-1 group O and P Vpu proteins do not counteract human tetherin

To evaluate anti-tetherin activity in the non-pandemic HIV-1 groups, we examined the ability of Vpu proteins from groups O and P to counteract human tetherin restriction (Figure 1). We used the Vpu proteins from viral isolates ANT70 and MVP5180, which are representative of group O subtypes I and II respectively [42-44], as well as the prototype group P isolate, RBF168 [17]. None of these Vpu proteins have previously been examined for anti-tetherin activity. As positive controls, we used Vpu proteins from HIV-1 group M (NL4-3) and N (YBF30) isolates [3]. The non-M Vpu proteins were constructed as EGFP fusion proteins to facilitate detection in the absence of specific or cross-reacting antibodies. Expression of each protein was confirmed by Western blotting, and functionality was demonstrated by the ability to degrade CD4 [38-41]. We found that all of the Vpu proteins reduced steady state CD4 levels, including the group N protein from isolate YBF30, which has previously been reported to be unable to remove CD4 from the cell surface (Figure 1B) [3].

Activity against human tetherin was assessed as the ability of the Vpu proteins to promote the release of HIV-1 virus like particles (VLPs) from HeLa cells, which naturally express tetherin [1,2]. Both group M and N Vpu demonstrated anti-tetherin activities, resulting in approximately 10-fold increases in the amount of VLPs released (Figure 1C). In contrast, neither the group O nor P proteins had any effect on VLP release. These data confirm and extend the findings about group O Vpu reported by Sauter *et al*. (2009) [3] and additionally reveal that HIV-1 group P Vpu has no activity against human tetherin.

### A group O proviral clone does not counteract human tetherin

**Figure 2:**
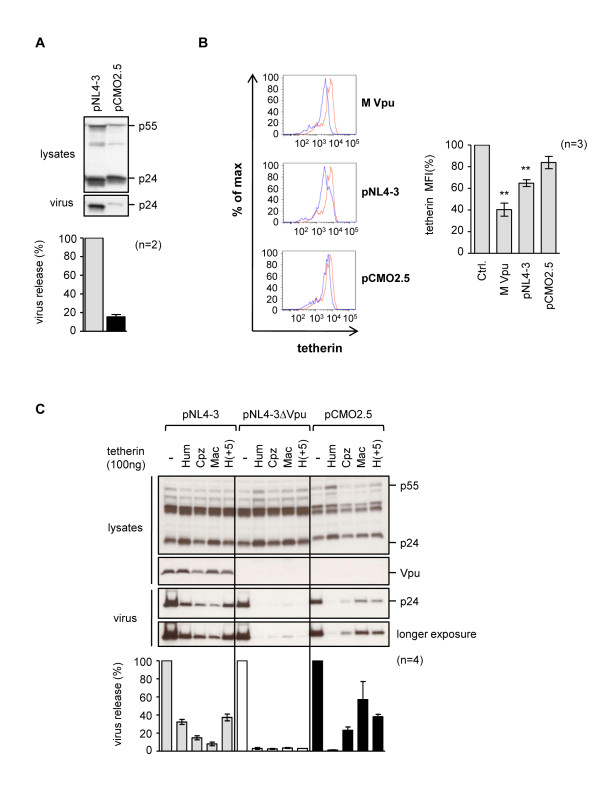
**Anti-tetherin activity in group O proviral clone pCMO2.5**. **(A) **Five μg of group M (pNL4-3) or group O (pCMO2.5) proviral clones were transfected into HeLa cells, and cell lysates and supernatants harvested and analyzed by Western blotting with an anti-p24 antibody. The percent virus release was calculated as the ratio of p24-reacting bands in the supernatants relative to the cell lysates, and normalized to 100% for the virus release from pNL4-3, for n = 2 independent experiments. **(B) **HeLa cells were transfected with 500 ng of a GFP expression plasmid alone (red), or together with 2 μg of either an expression plasmid for group M Vpu, or 5 μg of the proviral clones pNL4-3 or pCMO2.5 (blue). Cells were stained with an anti-tetherin antibody and analyzed for cell surface tetherin expression by FACS. The histograms show relative cell numbers (% of maximum) vs. tetherin expression (fluorescence intensity of APC) in cells gated for GFP expression; graph shows mean MFI in GFP-positive populations for n = 3 independent experiments, *p *< 0.01 (**). **(C) **Human (Hum), chimpanzee (Cpz), macaque (Mac), or a chimeric human tetherin, H(+5), containing an insert from Cpz-tetherin in the cytoplasmic tail, were transiently expressed in 293A cells, together with proviral clones pNL4-3, pNL4-3ΔVpu or pCMO2.5. The percent virus release was calculated as described above and made relative to the no tetherin control for each virus, for n = 4 independent experiments. The Vpu antisera used does not cross-react with the group O protein.

The lack of anti-tetherin activity we observed in the group O Vpu proteins led us to investigate whether we could detect any activity in a full-length replication competent group O clone, pCMO2.5 [45]. We measured the extent of virus release when pCMO2.5, or a control group M proviral clone, pNL4-3, were transfected into HeLa cells and found that pNL4-3 was approximately 5 times more efficient at releasing HIV-1 particles into the culture supernatant than pCMO2.5 (Figure 2A). This contrasted with the situation when the clones were transfected into 293A cells, which do not express significant amounts of tetherin [7,46], and where the virus release efficiency was found to be more equivalent (Figure 2C).

Previously, we and others have shown that tetherin antagonism by Vpu results in removal of tetherin from the cell surface [2,46-50]. We examined the tetherin population at the surface of HeLa cells following transfection by group M Vpu, pNL4-3 or pCMO2.5. FACS analysis revealed efficient tetherin removal by both group M Vpu and pNL4-3, but no significant change occurred with pCMO2.5 (Figure 2B). Together, these data suggest that the group O virus pCMO2.5 does not express a protein that has activity against human tetherin.

We next asked whether pCMO2.5 had activity against primate tetherins, which could have been retained from an ancestral virus, by examining the effects of human, chimpanzee and macaque tetherin on pCMO2.5 release. We also included a chimeric human tetherin, H(+5), that contains an insertion of the sequence DDIWKK from the cytoplasmic tail of chimpanzee tetherin, and which we have previously shown renders human tetherin susceptible to SIVmac Nef [7]. As controls we included pNL4-3 and a derivative pNL4-3ΔVpu, which does not express Vpu. Analysis of virus release from all three clones revealed that the NL4-3ΔVpu virus had no activity against any of the tetherins, while the wild type pNL4-3 virus had equal activity against the human and H(+5) tetherins, a partial activity against chimpanzee tetherin, as has previously been reported for HIV-1 Vpu [7,12], and only a small activity against macaque tetherin. In contrast, pCMO2.5 had no activity against human tetherin but was active against the other three proteins (Figure 2C). These findings suggest that this group O virus evolved from an ancestor that had activity against tetherin in primate hosts, and while it still retains some ability to counteract primate tetherins, it has not developed a comparable activity against human tetherin.

### Evidence for ancestral anti-tetherin activities in group O and P Nef proteins

**Figure 3:**
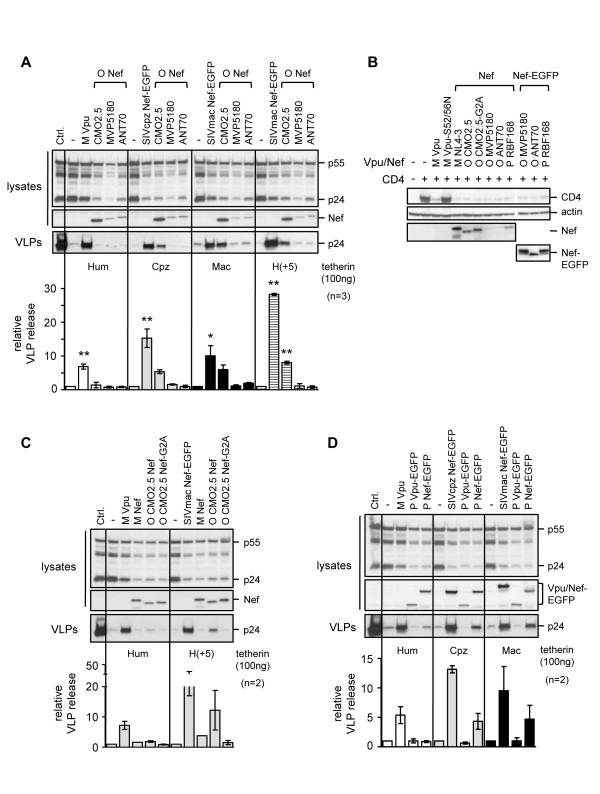
**Anti-tetherin activities in group O and P Nef proteins**. **(A) **Anti-tetherin activity of group O Nef proteins against the indicated tetherins was examined in 293A cells. Graph shows VLP release in the presence of indicated tetherins and Vpu or Nef proteins relative to the baseline levels of release from the tetherin alone controls (-), for n = 3 independent experiments. Group M Vpu, SIVcpz Nef-EGFP and SIVmac239 Nef-EGFP proteins were included as positive controls. Nef proteins were detected using antiserum raised against group M Nef protein that cross-reacts with group O proteins but not SIVmac Nef. Statistical significance is indicated as *p *< 0.05 (*) or *p *< 0.01 (**). **(B) **Human CD4 expression plasmid (1 μg) was transfected into 293A cells, together with 1 μg of the indicated Vpu or Nef plasmids. Group M Vpu and the defective Vpu-S52/56N mutant were included as positive and negative controls for CD4 degradation, respectively. Untagged Nef proteins were probed using anti-group M Nef antiserum and GFP-tagged Nef proteins were detected using anti-GFP antibody. **(C) **Activity of CMO2.5 Nef and a myristoylation site mutant (CMO2.5 Nef-G2A) against human and H(+5) tetherin, in 293A cells. Group M Vpu and SIVmac Nef were included as positive controls and group M Nef was included as a negative control. **(D) **Effect of group P Vpu or Nef proteins on HIV-1 VLP release in the presence of different tetherins, measured in 293A cells, as previously described, for n = 2 independent experiments. Vpu and Nef expression was detected using anti-GFP antibody.

The fact that the H(+5) tetherin was antagonized by pCMO2.5 implicated Nef as the anti-tetherin factor in this virus. We therefore examined the activity of the pCMO2.5 Nef protein against the panel of tetherin proteins (Figure 3A). We also included Nef proteins from HIV-1 O isolates ANT70 and MVP5180, since the Vpu proteins from these viruses also lacked activity against human tetherin (Figure 1C). As positive controls we included group M Vpu, and the Nef proteins from SIVcpz and SIVmac239, which are able to counteract the human, chimpanzee and macaque tetherins, respectively [3-5,7]. We noticed that all cells transfected with Nef expression plasmids displayed lower levels of intracellular HIV-1 Gag proteins. Although we have no explanation for this consistent observation, the use of a virus release assay that is based on the ratio of p24-reacting proteins in the supernatant versus cell lysates, allows us to control for such effects and still measure the effect of tetherin, and its antagonists, on the efficiency of virus release.

Analysis of VLP release in the presence of the various tetherins revealed that pCMO2.5 Nef had activity against chimpanzee, macaque and H(+5) tetherin, but not human tetherin. In contrast, the other two group O Nef proteins had no activity against any of the tetherins examined (Figure 3A). Since detection of some of the group O/P Nef proteins on Western blots by the anti-group M Nef antibody was not robust, we also constructed Nef-EGFP fusion proteins, and confirmed their expression by Western blotting with an anti-GFP antibody to rule out problems with protein stability or expression (Figure 3B). Using the EGFP-tagged proteins, we observed the same results as with the untagged proteins (data not shown). Finally, we confirmed the activity of all Nef constructs, both untagged and EGFP-tagged, using a CD4 degradation assay [40,51] (Figure 3B).

We further investigated the activity of pCMO2.5 Nef by introducing a G2A substitution that prevents Nef myristoylation and plasma membrane localization [45]. A similar substitution in SIVmac239 Nef has been shown to block its anti-tetherin activity [4]. Following this mutation, pCMO2.5 Nef lost activity against H(+5) tetherin (Figure 3C). Together these data suggest that the partial activity the pCMO2.5 virus has against primate tetherins is a function of its Nef protein.

Next, we examined whether the Nef or Vpu proteins from the group P isolate, RBF168 [17], had anti-tetherin activity. We observed the same pattern as with pCMO2.5, finding no activity against human tetherin, but partial activity in the group P Nef protein against both macaque and chimpanzee tetherins (Figure 3D). Group P Nef, either untagged or EGFP-tagged, was able to degrade human CD4 (Figure 3B). Together, these data suggest that the group P viruses have also evolved from an ancestor that used the Nef protein to counteract tetherin in its primate hosts, but similar to the group O viruses, have failed to adapt to counteract human tetherin.

### Lack of anti-tetherin activity in group O Vpu maps to the TM domain

**Figure 4:**
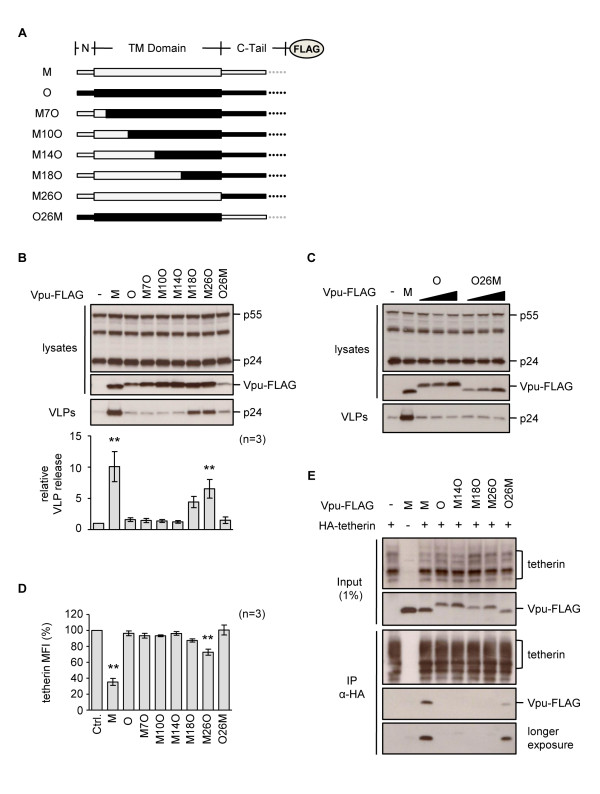
**Characterization of chimeric M-O Vpu proteins**. **(A) **Schematic (not to scale) of major domains in FLAG-tagged chimeric Vpu proteins formed between the functional group M (NL4-3, grey) and non-functional group O (ANT70, black) proteins. Numbers in name indicate junction site and refer to the group M residues. **(B) **Activity of M-O chimeric Vpu-FLAG proteins against human tetherin in HeLa cells. Relative VLP release was calculated as described previously and is shown for n = 3 independent experiments, *p *< 0.01 (**). Expression of Vpu-FLAG proteins was confirmed using an anti-FLAG antibody. **(C) **Vpu-FLAG proteins O and O26M are expressed at lower levels than other Vpu constructs, so increasing amounts of the plasmids were transfected into HeLa cells (range 2 to 6 μg), to confirm that their lack of anti-tetherin activity was not simply due to lower levels of expression. As a control, 2 μg of group M Vpu-FLAG was transfected. **(D) **Ability of chimeric M-O Vpu-FLAG proteins to remove tetherin from the surface of HeLa cells. Cells were co-transfected with 2 μg of indicated Vpu plasmid and 500 ng of GFP expression plasmid and MFI calculated in the GFP-positive population. Graph shows mean MFI for n = 3 independent experiments, *p *< 0.01 (**). **(E) **293T cells were transfected with HA-tagged tetherin alone (500 ng) or together with the indicated Vpu-FLAG expression plasmids (1 μg), except O and O26M (2 μg). Immunoprecipitation (IP) was performed using anti-HA MicroBeads, followed by Western blot analysis of both input lysates (1%) and immunoprecipitates, using anti-FLAG and anti-tetherin antibodies.

We next examined why the group O Vpu proteins did not have activity against human tetherin. We constructed a series of FLAG-tagged M-O chimeras between the Vpu proteins from NL4-3 and ANT70 (Figure 4A), confirmed their expression by Western blotting, and analyzed their ability to counteract human tetherin in a VLP release assay (Figure 4B). To rule out problems due to the lower expression of constructs O and O26M, we also increased the amounts of DNA transfected into HeLa cells to give equivalent levels of Vpu expression as the functional group M protein (Figure 4C). We identified as important the first 18 amino acids of group M Vpu, since chimera M18O had some activity, but M14O did not. Increasing the amount of M sequences to contain the full TM domain (M26O) further increased tetherin antagonism. Although the TM domain of group M Vpu has been shown to be a key determinant of the specificity of tetherin antagonism [6], a role for a hinge region and two alpha helices in the cytoplasmic domain of Vpu has also been noted [52]. The activity of M26O suggests that the cytoplasmic tail of ANT70 group O Vpu is functional for this activity.

The ability of the chimeras to remove human tetherin from the surface of HeLa cells was also examined (Figure 4D). Only the wild-type group M Vpu was able to markedly remove tetherin from the cell surface, with the M26O chimera also showing an effect. For the minimal functional chimera, M18O, its expression consistently reduced tetherin levels but this did not reach statistical significance, which may explain its less efficient ability to antagonize tetherin.

Group M Vpu has been shown to physically interact with human tetherin by co-immunoprecipitation (co-IP) [47,48,53-55]. We examined the ability of the panel of chimeric proteins to co-IP with an HA-tagged tetherin, and found that only group M Vpu, and to a lesser extent the O26M chimera, was able to demonstrate such an interaction (Figure 4E). The lack of interaction between tetherin and either of the functional chimeras, M18O or M26O, was surprising, but may reflect a less than optimal interaction that is not detected in this system. More unexpected was the positive interaction observed between tetherin and the non-functional O26M chimera. This suggests that a physical interaction between tetherin and Vpu can occur in the absence of a functional tetherin antagonism, and may implicate other partners or processes in tetherin counteraction.

**Figure 5:**
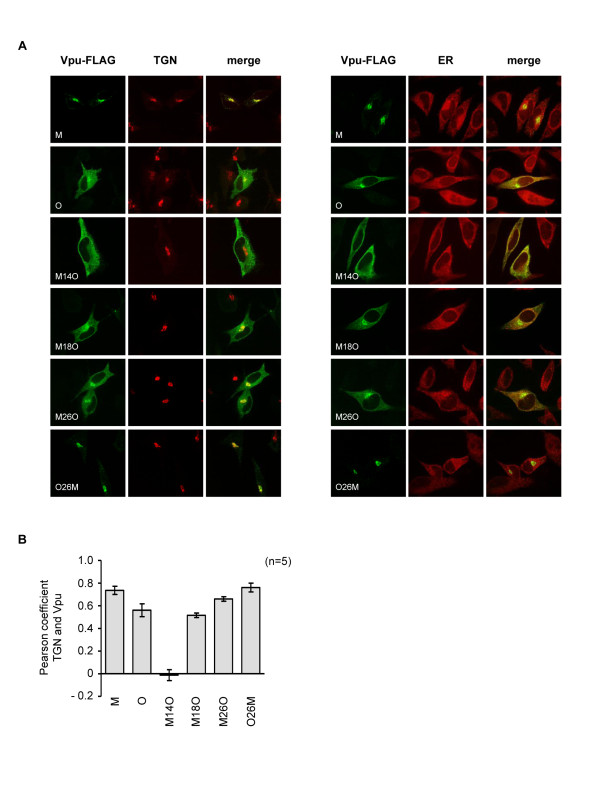
**Subcellular localization of chimeric M-O Vpu proteins**. **(A) **Subcellular localization of Vpu chimeras in HeLa cells, transfected with the indicated Vpu-FLAG chimeras and stained with antiserum against FLAG (green), and TGN (left) or ER (right) markers (red). **(B) **The degree of co-localization of Vpu proteins with the TGN marker was calculated using the Pearson coefficient.

A significant fraction of group M Vpu is present in the TGN [46,56], and Vpu co-expression further concentrates tetherin to this compartment [46,48]. We considered the possibility that the difference between the functional and non-functional M-O chimeras could reflect differences in their cellular distribution. Using confocal microscopy, we observed that the group M and O Vpu proteins had distinct distributions, with the group M protein showing a strong colocalization with the TGN, while the group O protein was found concentrated in the TGN, but also had a more reticular distribution and ER overlap (Figure 5A). The M-O chimeras had various distributions, being either predominantly in the TGN (O26M), excluded from the TGN (M14O), or present in both the TGN and ER (the rest of the chimeras). We found no pattern that easily explained the functionality, or lack thereof, of the chimeras (Figure 5B). However, comparison of the non-functional M14O and the partially functional M18O chimera revealed re-acquisition of a TGN distribution in M18O (Figure 5B), suggesting that while TGN localization is not sufficient for anti-tetherin activity, it may well be necessary.

### Alanine-18 is important for group M Vpu localization and tetherin-Vpu interactions

**Figure 6:**
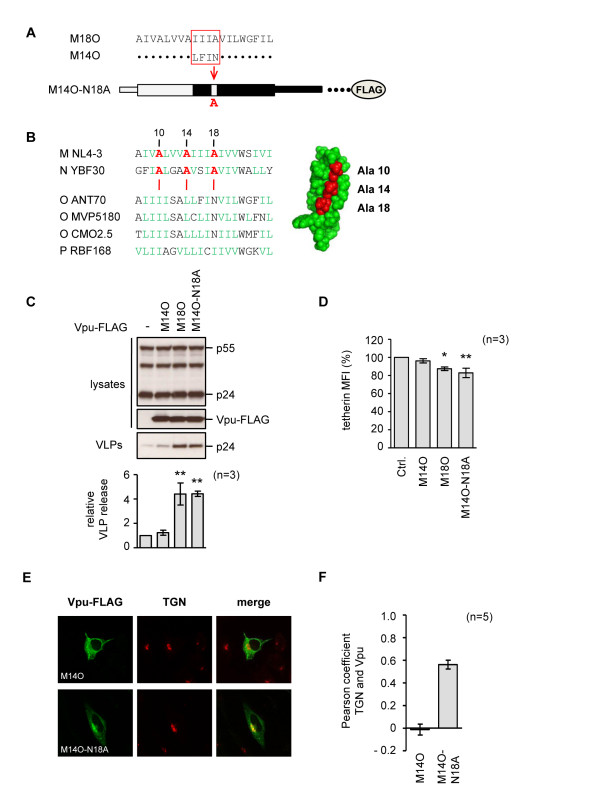
**Role of Alanine-18 in tetherin antagonism by M-O chimeric Vpu proteins**. **(A) **Schematic of TM domains from M14O and M18O, highlighting location of alanine-18, and configuration of M14O-N18A. **(B) **Sequence alignment of Vpu TM domains from indicated viruses, with numbering based on group M protein. Alanine residues that are conserved in the functional group M and N Vpu proteins, but are absent in the non-functional group O and P proteins, are labeled in red; non-aromatic hydrophobic residues are labeled in green. Also shown is the 3-D structure of the group M Vpu TM domain (residues 7 to 25 from isolate BH10) [57], created using PyMOL software (Schrödinger LLC), with the conserved alanine residues highlighted in red. **(C) **Effects of indicated Vpu proteins on HIV-1 VLP release from HeLa cells, measured as previously described, *p *< 0.01 (**). **(D) **Effects of indicated Vpu proteins on cell surface tetherin in HeLa cell, measured as previously described, *p *< 0.05 (*) or *p *< 0.01 (**). **(E) **Subcellular localization of M14O and M14O-N18A proteins in HeLa cells, detected by confocal microscopy. Vpu proteins were visualized using anti-FLAG antibody (green), and the TGN (red) was detected with specific antisera. **(F) **The degree of co-localization of Vpu proteins with the TGN marker was calculated using the Pearson coefficient.

The functional M18O and non-functional M14O Vpu protein differ at three amino acids (Figure 6A). We were particularly interested in alanine-18 in the group M sequence, since this is part of a string of alanines that form a diagonal face of the transmembrane helix of Vpu [57]. Furthermore, this face is conserved in both the functional group M and N Vpu proteins, but is not present in the group O or P proteins (Figure 6B) [54,55]. We found that the introduction of alanine-18 into chimera M14O (designated M14O-N18A) was sufficient to confer anti-tetherin activity (Figure 6C) and remove tetherin from the cell surface (Figure 6D). In addition, alanine-18 altered the cellular distribution of the chimera, increasing its co-localization with the TGN compartment (Figure 6E, F)

**Figure 7:**
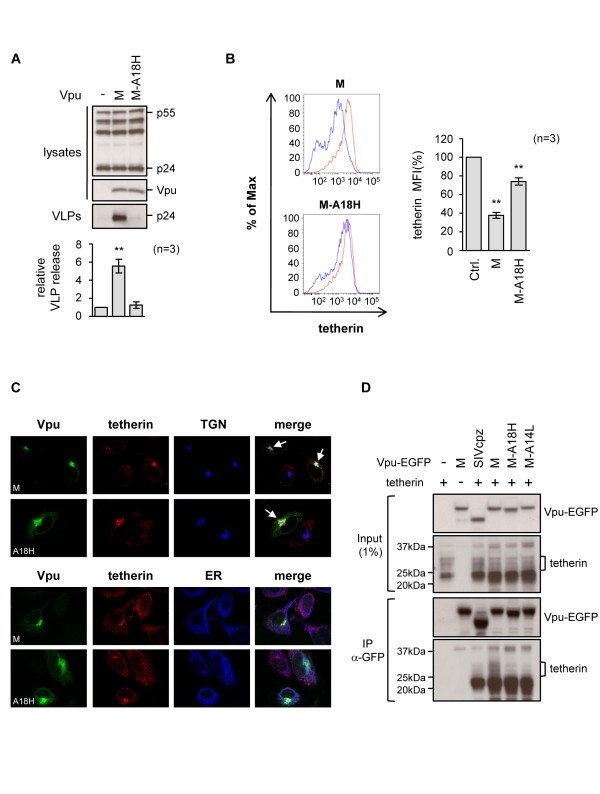
**Alanine-18 is important for group M Vpu anti-tetherin activity**. **(A) **Effect of group M Vpu and the A18H mutant on VLP release from HeLa cells, measured as previously described, *p *< 0.01 (**). **(B) **Effect of Vpu on tetherin expression on cell surface of HeLa cells, in the absence (red) or presence (blue) of Vpu, examined as previously described. *p *< 0.01 (**) **(C) **Subcellular localization of indicated Vpu constructs in HeLa cells and their effects on tetherin distribution. TGN (top) and ER (bottom) markers are included. Arrows indicate cells expressing Vpu that redistributed tetherin to the TGN. **(D) **293T cells were transfected with tetherin alone (500 ng) or together with the indicated Vpu-EGFP expression plasmids (5 μg), where M is the Vpu protein from group M strain HXB2. SIVcpz Vpu-EGFP, which is not active against human tetherin, and a Vpu mutant, A14L, were included as negative controls. Immunoprecipitation (IP) was performed using anti-GFP MicroBeads, followed by Western blot analysis of both input lysates (1%) and immunoprecipitates, using either anti-GFP or anti-tetherin antibodies. Mature glycosylated forms of tetherin run between 25 and 37 kDa (bracketed) and interact specifically with wild-type group M Vpu, while an immature faster running species that is also present in the transfected cells is non-specifically pulled down by all Vpu proteins.

Further evidence for the importance of alanine-18 was obtained by investigating the A18H mutant of group M Vpu [58]. In agreement with a recent report [55] we observed no functional anti-tetherin activity for this mutant (Figure 7A), although it did possess a partial ability to reduce tetherin levels on the cell surface (Figure 7B). It has been reported that A18H has a different cellular distribution than the wild-type protein, being present in the ER [55]. We also noted a more reticular, ER localization for the A18H mutant as well as being in the TGN (Figure 7C). In addition, the A18H mutant was reported not to co-localize with tetherin [55]. However, our experiments produced a somewhat different finding, since we observed that the A18H mutant retained a significant ability to redistribute tetherin to the TGN in about 75% of the cells examined (Figure 7C, arrowed), although 25% of the cells did not redistribute tetherin in this way.

It has recently been suggested that the alanine face of group M Vpu could serve as a direct binding surface for tetherin [54,55]. We examined whether we could also detect a specific Vpu-tetherin interaction, and whether alanine face mutations reduced this. Using EGFP-tagged wild-type M Vpu as a positive control, and the SIVcpz Vpu and a previously described non-interacting Vpu mutant (A14L) as negative controls [3,7,54], we found that we were able to specifically detect interactions between Vpu and the mature glycosylated forms of tetherin that run between 25 and 37kD [59], although the faster-running immature forms of tetherin that are a major species in transfected 293T cells were non-specifically immunoprecipitated in all cases. Using this system, we found that both mutations A18H and A14L prevented co-immunoprecipitation (Figure 7D). We conclude that the A18H mutation perturbs an essential interaction between Vpu and tetherin, resulting in reduced sequestration of tetherin in the TGN, less efficient removal of tetherin from the cell surface and an inability to counteract the restriction of virus release.

### Alanine face residues are not sufficient to confer anti-tetherin activity to group O Vpu

**Figure 8:**
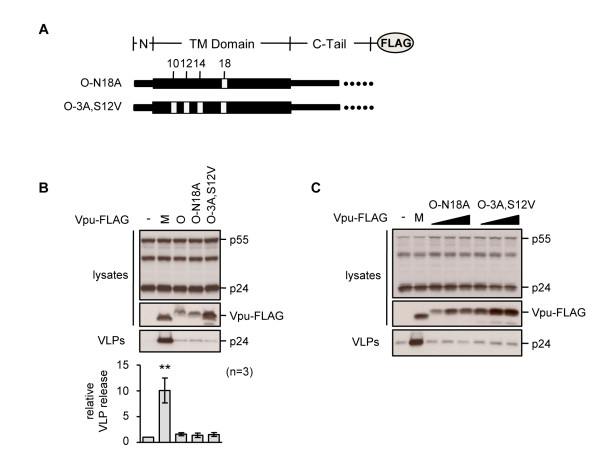
**Residues from group M Vpu are not sufficient to confer anti-tetherin activity to group O Vpu**. **(A) **Schematic of Vpu-FLAG proteins based on group O Vpu, with group M substitutions at positions 10, 12, 14 and 18. **(B) **Ability of indicated Vpu proteins to increase HIV-1 VLP release from HeLa cells, measured as previously described, *p *< 0.01 (**). **(C) **Effect of transfecting increasing amounts of plasmids O-N18A and O-3A,S12V (range 2 to 6 μg) on VLP release from HeLa cells.

We next examined whether the alanine face residues present in group M Vpu were sufficient to confer recognition of human tetherin to group O Vpu by substituting either alanine-18 alone (O-N18A), or the combination of three alanines at positions 10, 14 and 18 together with serine at 12 to valine (O-3A,S12V), which more fully mimics the group M TM domain in this region (Figure 8A). However, we found that neither alteration conferred anti-tetherin activity to the group O protein (Figure 8B, C). These data suggest that additional sequences present in the first 14 residues of group M Vpu are also required to confer anti-tetherin activity.

## Discussion

To date, three different proteins in the primate lentiviruses, Vpu, Env and Nef, have been found to target the BST-2/tetherin protein [1-11]. Together with the host species specificities displayed by these factors, this suggests that a strong selective pressure exists to counteract tetherin, and that such adaptations have been important enablers of host species expansion. HIV-1 is currently recognized to exist in four different groups; group M, which is the major driver of the worldwide pandemic, and the minor groups N, O and P. It has previously been reported that while the Vpu proteins from both groups M and N have activity against human tetherin, neither the Vpu or Nef proteins from group O viruses have such a function [3,60]. Our study has confirmed these findings and further demonstrated that a recently described group P virus also has no activity against human tetherin in either its Vpu or Nef proteins.

Despite the apparent lack of anti-human tetherin activity in group O Vpu and Nef proteins, group O viruses are clearly still pathogenic in humans. They were previously reported to have been responsible for 20.6% of infections in Cameroon in the 1986-1988 time period although this dropped to only about 1.4% by 1997 [28]. We, therefore, considered the possibility that another group O protein, apart from Vpu or Nef, could have taken over an anti-tetherin function, similar to the use of the Env protein by HIV-2 [9-11]. However, examination of the Env and Vpr proteins from the pCMO2.5 infectious molecular clone of HIV-1 O found no evidence of such an activity (data not shown), and the whole virus was restricted by human tetherin. These data point to a lack of anti-tetherin activity in HIV-1 group O viruses, and further suggest that the lower prevalence of group O in human populations could derive, in part, from their inability to counteract human tetherin.

As an alternative explanation, it is possible that group O and P viruses do not encode an anti-tetherin factor because infection by these viruses does not induce tetherin. It has previously been reported that infection by SIVagm only transiently induces type I interferon in the early stages of an infection [61], and such limited production would be expected to limit the expression of tetherin. A corresponding lack of anti-tetherin factors in the genome of this virus has been reported [62]. However, other reports have described anti-tetherin activities in the Env and Nef proteins of SIVagm [5,8], so it is unclear if such a precedent exists. Therefore, while it is possible that the group O and P viruses could avoid tetherin restriction indirectly, for example through low interferon induction, further studies would be needed to determine if this is indeed the case.

By analyzing activity against a panel of tetherins, we found that pCMO2.5 retained a residual anti-tetherin activity in its Nef protein that was able to recognize both primate tetherins and a modified human protein with an insert (DDIWKK) in its cytoplasmic tail. This motif is also required for the interaction of SIV Nef proteins with primate tetherins [3-7], and suggests a similar mechanism. These observations are consistent with the immediate ancestor of HIV-1 group O using Nef to counteract tetherin in its primate host, but being unable to adapt Nef to counteract the human protein that is missing this cytoplasmic target motif. Transmission of HIV-1 group M and HIV-2 to humans has resulted in the adaptation of Vpu and Env, respectively, to counteract human tetherin. The HIV-1/SIVcpz lineage of primate lentiviruses originated from a recombination between SIVrcm and the SIVmus/mon/gsn sub-lineage [63,64], and modern day SIVmus/mon/gsn viruses possess anti-tetherin activities in Vpu. Indeed, we have even detected partial activity against human tetherin in an SIVgsn isolate [7]. Therefore, adaptation of Vpu to human tetherin would seem to be a straightforward solution for all HIV-1 viruses, and it is somewhat surprising that the group O and P viruses do not appear to have evolved this activity. Whether this reflects some other demand on Vpu function in these viruses that precludes such evolution is an open question.

We used the finding that the group O Vpu has not adapted to counteract human tetherin to investigate domains in group M Vpu that are necessary for its activity. Previous studies of group M Vpu have suggested three domains in the protein that are involved in tetherin antagonism; the TM domain [1,2], a membrane proximal positively charged hinge region in the cytoplasmic tail [52] and a β-TrCP binding motif based on two conserved serine residues in the tail that is also required for Vpu's ability to degrade CD4 [2,47,49,53]. The residues in the membrane proximal charged region are not conserved between groups M, O and P, while two and one of the tail serines, respectively, are present in the group O and P proteins. All group O and P Vpu proteins used in this study retained the ability to degrade CD4, suggesting that these serine motifs are still able to recruit β-TrCP or that the proteins use an alternate strategy to degrade CD4.

Through the construction of M-O Vpu chimeras, we ruled out any significant defect in the cytoplasmic tail of the group O protein used in these studies (ANT70), since the chimera M26O was fully functional. In contrast, a series of chimeras with junctions in the TM domain identified as important the first 18 residues of group M Vpu, and in particular highlighted a role for alanine-18. Our findings differ from a recent study using group O isolates MVP9435 and HJ001, which mapped the minimal contribution of group M sequences that could produce a functional M-O chimera as comprising both the full TM domain and the hinge region of the cytoplasmic tail [60]. However, our data do agree with another conclusion from this study, that the group O cytoplasmic tail promotes the ER localization of the protein [60]. Indeed, the different cellular distribution of the group O Vpu protein compared to group M may reflect a requirement of another function of Vpu, which makes the protein no longer compatible with tetherin antagonism in a human host.

Residue alanine-18 is part of a helical face of conserved alanine residues that are found in the group M and N proteins but not in group O or P, and could therefore represent a protein interacting domain that recruits tetherin or some additional factor. A defective group M Vpu with the equivalent of an A18H substitution has previously been described [55,58]. We confirmed the lack of anti-tetherin activity in this mutant and further investigated the basis for its defect by analyzing both its sub-cellular distribution, and its ability to remove tetherin from the cell surface and concentrate it in the TGN, as previously observed for the wild type group M Vpu [46,48]. We found that the A18H substitution resulted in a protein that was still located in the TGN, although it was more present in the ER than the wild-type protein. Co-immunoprecipitation analyses also revealed that this mutation prevented a specific Vpu-tetherin interaction. Interestingly, the A18H mutant was still able to concentrate tetherin in the TGN in the majority (75%) of cells in which it was expressed, and it displayed some ability to remove tetherin from the cell surface, but it is likely that these activities are below a threshold needed to overcome tetherin restriction.

We also noted that the introduction of alanine-18 into the defective chimera, M14O, to create construct M14O-N18A, was sufficient to re-locate the protein from an ER-like distribution to the TGN, and to restore activity against tetherin. However, substitution of either alanine-18 alone, or a more extensive stretch of residues that include the alanine face comprising residues 10, 14 and 18 into the group O protein was not sufficient to confer activity, implicating additional residues in the TM of group M Vpu.

Overall, the data from the M-O chimeras argue against a simple model whereby Vpu location in the TGN, and the ability to co-immunoprecipitate with tetherin, is predictive of anti-tetherin activity. For example, although comparison of the M14O and M14O-N18A proteins indicates that the presence of Vpu in the TGN is necessary to counteract tetherin, the lack of activity of the O26M chimera, which has a robust presence in the TGN compartment, suggests that this is not sufficient. Similarly, although we were able to detect co-immunoprecipitation between tetherin and group M Vpu that required the presence of alanine-18, as previously reported [55], we were unable to detect an association between tetherin and the functional M18O and M26O chimeras, while being able to detect such an interaction with the defective O26M chimera. Although these observations may reflect either the technical limitations of co-immunoprecipitation assays, or defects that are specific for this series of chimeric proteins, it is also possible that additional factors are involved in the Vpu-tetherin interaction that have not yet been uncovered.

## Conclusions

Primate lentiviruses display a remarkable variation in the strategies they use to target the BST-2/tetherin protein. Despite the seeming importance of this interaction, the HIV-1 group O and P viruses do not possess anti-tetherin activities in their Vpu or Nef proteins that recognize human tetherin, and studies with a group O proviral clone further suggest that they may not have been able to evolve such an activity. Such a failure to counteract human tetherin may have impeded their spread in human populations. Studies with chimeric proteins suggest that multiple changes would be required in the group O Vpu protein to acquire such an activity, including an alteration in the protein's sub-cellular distribution, and this may have produced too high a barrier to overcome.

## Methods

### Cell lines

HeLa, 293T and LLC-MK2 cells were obtained from the American Type Culture Collection; 293A cells were obtained from Qbiogene/MP Biomedicals (Irvine, CA). All cells were maintained in D10 medium: Dulbecco's modified Eagle's medium (DMEM) (Mediatech, Herndon, VA) supplemented with 10% fetal bovine serum (FBS) (Mediatech) and 2 mM glutamine (Gemini Bio-Products, West Sacramento, CA).

### Plasmids

Plasmid pHIV-1-pack expresses HIV-1 Gag-Pol and Rev [11] and is used to produce HIV-1 virus-like particles (VLPs). Plasmid pcDNA-Vphu (renamed M Vpu) encodes a human codon-optimized form of Vpu from group M HIV-1 isolate NL4-3, and was kindly provided by Klaus Strebel (NIH). A Vpu mutant defective in CD4 downregulation was generated by changing serine residues at 52 and 56 to asparagines (M Vpu-S52/56N) by site-directed mutagenesis [65] and a substitution of alanine-18 to histidine was made to generate plasmid M Vpu-A18H. Vpu sequences from HIV-1 group N YBF30 (GenBank: CAA06815), group O strains ANT70 (AAA99882) and MVP5180 (AAA44863), and group P strain RBF168 (ACT66828) were synthesized as human codon-optimized open-reading frames (Bio Basic Inc., Ontario, Canada). EGFP-tagged versions of the proteins were constructed by cloning into vector pAcEGFP-N1 (Clontech, Mountain View, CA). An expression plasmid for SIVcpz GAB1 Vpu-GFP [66] was provided by Ed Stephens (University of Kansas). FLAG-tagged chimeric group M-O Vpu proteins were made by splice-overlap PCR between group M (NL4-3) and group O (ANT70) proteins and cloned into vector pCMV6-XL5 (Origene, Rockville, MD); nomenclature follows the convention where chimera M7O has the first 7 amino acids of group M Vpu fused to the rest of the group O Vpu. Additional point mutations were introduced by site-directed mutagenesis.

Proviral clone pNL4-3 was obtained through the AIDS Research and Reference Reagent Program (ARRP), from Dr. Malcolm Martin [67]; derivative pNL4-3ΔVpu was generated by PCR mutagenesis and contains a deletion of the first 10 nucleotides of the Vpu open-reading frame. The full-length group O proviral clone pCMO2.5 [45] was provided by Hans-Georg Kräusslich (University of Heidelberg, Germany).

Nef proteins from pNL4-3 and pCMO2.5 were PCR amplified from the proviral clones and cloned into vector pAcEGFP-N1, with the addition of a stop codon to prevent expression of the EGFP tag. A Gly to Ala mutation at the second amino acid was introduced into CMO2.5 Nef by site-directed mutagenesis (CMO2.5 Nef-G2A). Nef sequences from HIV-1 group O ANT70 (AAA99884), MVP5180 (AAA44865), and group P RBF168 (ACY40660) were synthesized (Bio Basic Inc.) and cloned into vector pAcEGFP-N1 to generate Nef-EGFP tagged proteins; untagged versions were created by introducing stop codon after the Nef sequence. Nef-EGFP proteins from SIVmac239 and SIVcpz EK505 have been previously described [7].

Expression plasmids for BST-2/tetherin from human (Hum), chimpanzee (Cpz) and human tetherin with the sequence DDIWKK replacing amino acid E-14 (H+5), have been previously described [7]. Full-length macaque tetherin (Mac) was PCR amplified from LLC-MK2 cells and cloned into vector pCMV6-XL5 (Origene). Human tetherin with an HA tag at the N-terminus (HA-tetherin) was PCR amplified and cloned into vector pCMV6-XL5 (Origene). Human CD4 was amplified from plasmid CD4-YFP, provided by Stefano Marullo **(**Université Paris Descartes, France) [68] and cloned into vector pCMV6-XL5.

### Production and analysis of HIV-1 VLPs

HIV-1 VLPs were generated from HeLa and 293A cells by transient transfection as previously described [7]. The following amounts of plasmid DNA were used: 2 μg Vpu constructs, 5 μg proviral clones, 0.6 μg Nef constructs, 100 ng tetherin constructs. Amounts of DNA in all transfections were equalized using empty expression vectors. Cell lysates and viral particles were collected 24 hours post-transfection and the levels of p24 protein in both lysates and supernatants analyzed by Western blot, as previously described [7], using rabbit HIV-1SF2 p24 antiserum (ARRRP) at a 1:3,000 dilution, followed by horseradish peroxidase (HRP)-conjugated goat anti-rabbit IgG (1:10,000) (Santa Cruz Biotechnology, Santa Cruz, CA). Specific proteins were visualized using the enhanced chemiluminescence (ECL) detection system (Amersham International, Arlington Heights, IL). Exposed and developed films were scanned and quantified using the public domain NIH ImageJ software. The fold-enhancement of virus release was calculated as the ratios of p24-reacting bands in supernatants:lysates, and normalized to either the pHIV-1-pack only control, or pHIV-1-pack plus tetherin, as indicated. The statistical significance of data was determined using one-way analysis of variance (ANOVA) followed by Dunnett's multiple comparison test from GraphPad Prism (GraphPad Software, La Jolla, CA).

### Western blotting

Expression of Vpu-EGFP and Nef-EGFP constructs was detected by Western blotting of cell lysates using rabbit anti-GFP at a 1:1000 dilution (Invitrogen, Carlsbad, CA). CD4 expression was detected using rabbit anti-CD4 at a 1:1000 dilution (Santa Cruz Biotechnology Inc). Actin expression was detected using a monoclonal anti-actin antibody, at 1:3000 (Sigma-Aldrich, St. Louis, MO). Nef expression was detected with rabbit anti-Nef antiserum at a 1:1000 dilution (ARRRP, from Ronald Swanstrom) and Vpu expression was detected using rabbit anti-HIV-1 Vpu antiserum at a 1:2000 dilution (ARRRP, from Frank Maldarelli and Klaus Strebel). Expression of Vpu-FLAG proteins was detected using rabbit anti-FLAG at a 1:1000 (Sigma-Aldrich). Tetherin expression was confirmed by rabbit anti-tetherin antibody at a 1:10000 dilution (ARRRP, from Klaus Strebel). The secondary antibodies used were a 1:10,000 dilution of HRP-conjugated goat anti-rabbit IgG (Santa Cruz Biotechnology) and a 1:10,000 dilution of goat anti-mouse IgG (Sigma-Aldrich).

### Flow cytometry

HeLa cells were transfected with 500 ng of a GFP expression plasmid together with 2 μg of untagged Vpu expression plasmid or 5 μg of the proviral clones pNL4-3 or pCMO2.5. For the M-O chimeric Vpu-FLAG proteins, 2 μg of the plasmids were transfected in HeLa cells. Twenty-four hours later, cells were harvested, incubated in 1% bovine serum albumin/phosphate-buffered saline (PBS) for 20 minutes at 4°C, and stained for tetherin expression using rabbit anti-tetherin antiserum (ARRRP, from Klaus Strebel) at a 1:5,000 dilution for 20 minutes at room temperature, followed by washing three times with PBS and incubation with goat anti-rabbit IgG conjugated to Alexa Fluor 647 (Invitrogen) at a 1:300 dilution for 20 minutes at room temperature. After staining, cells were fixed with 4% paraformaldehyde at room temperature and analyzed with a BD FACSCanto II (BD Biosciences, San Jose, CA). Ten thousand events were collected and data analyses were performed using FlowJo 6.2 software (Tree Star, Ashland, OR).

### Confocal microscopy

HeLa cells plated in 10-cm dishes were transfected with 1 μg of Vpu or 2 μg of Vpu-FLAG expression plasmids, then 18-24 hours later, seeded on coverslips coated with poly-L-lysine (Sigma-Aldrich). The cells were incubated for an additional 24 hours at 37°C, fixed with 4% paraformaldehyde for 20 minutes at room temperature, washed in PBS, permeabilized for 10 mins in 0.1% Triton X-100 at room temperature, and washed again in PBS prior to antibody staining. Vpu was detected using either rabbit anti-Vpu antiserum (ARRRP) at a 1:1000 dilution or a rabbit anti-FLAG polycolonal antibody at a 1:500 dilution (Sigma-Aldrich). The TGN was labeled using sheep polyclonal anti-TGN46 antibody (Serotec, Oxford, UK) at a 1:1000 dilution and the ER with a goat polyclonal anti-calnexin antibody (Santa Cruz Biotechnology) at a 1:200 dilution. Tetherin was detected using a polyclonal mouse anti-tetherin antibody, MaxPab H00000684-B02P (Abnova, Taipei City, Taiwan), at a 1:250 dilution. The conjugated secondary antibodies used were donkey anti-mouse Alexa Fluor 594, donkey anti-sheep Alexa Fluor 647, and donkey anti-rabbit Alexa Fluor 488, and donkey anti-goat Alex Fluor 647 at 1:200 dilutions (Invitrogen). Processed cells were mounted in Prolong Gold antifade reagent (Molecular Probes, Invitrogen). Images were acquired with the PerkinElmer Ultraview ERS laser spinning disk confocal imaging system at 100× magnification (PerkinElmer, Waltham, MA) and processed using Volocity software (Improvision, PerkinElmer) and Adobe Photoshop Creative Suite 2. Co-localization analysis of confocal images was performed using NIH ImageJ software to calculate Pearson correlation coefficient, as described previously [46].

### Immunoprecipitation

293T cells in 10-cm dishes were transfected with 500 ng of untagged human tetherin, together with 5 μg of group M HXB2 Vpu-EGFP or the equivalent A18H mutant (corresponding to residue alanine-19 in HXB2), kindly provided by Ed Stephens [58]. An A14L mutant was generated by site-directed mutagenesis. Alternatively, for HA-tetherin pull-downs, 293T cells were transfected with 500 ng of HA tagged human tetherin (HA-tetherin) together with 2 μg of Vpu-FLAG proteins. Cell lysates were harvested 24 hours post-transfection and pull-downs performed using the μMACS GFP or the μMACS HA isolation kits (Miltenyi Biotech Inc., Auburn, CA), according to the manufacturer's protocol. Specific proteins in the input lysate and immunoprecipitates were detected by Western blotting using anti-GFP, anti-FLAG or anti-tetherin antisera, as described above.

## Competing interests

The authors declare that they have no competing interests.

## Authors' contributions

SJY participated in the design of the study, performed most of the experiments, and wrote the draft manuscript. LAL, CME and KGH contributed to experiments and participated in the review of the manuscript. PMC conceived and co-ordinated the study, and wrote the final manuscript. All authors read and approved the final manuscript.
